# Efavirenz-induced grade III gynecomastia in an HIV-positive male patient: a case report and literature review

**DOI:** 10.3389/fmed.2025.1574364

**Published:** 2025-09-18

**Authors:** Lu Zhou, Yunhao Luo, Weiwei Liao, Delin Yang, Wen Hu

**Affiliations:** ^1^Department of Breast Surgery, First People’s Hospital of Liangshan, Xichang, China; ^2^Department of Critical Care Medicine, Chengdu Integrated Traditional Chinese Medicine and Western Medicine Hospital, Chengdu, China; ^3^Department of Breast Surgery, West China Tianfu Hospital, Sichuan University, Chengdu, China

**Keywords:** efavirenz, antiretroviral therapy, gynecomastia, HIV, psychological distress, case report

## Abstract

**Background:**

Efavirenz, a non-nucleoside reverse transcriptase inhibitor, is a preferred drug for the treatment of HIV-positive patients. However, one of its adverse effects is gynecomastia, which not only causes psychological distress to patients, affecting their daily lives and work, but also may undermine their treatment adherence. Although numerous studies have documented cases of gynecomastia in HIV-positive patients after antiretroviral therapy (ART), severe cases of gynecomastia are rarely reported.

**Patient presentation:**

A 29-year-old male patient initiated a triple therapy regimen of efavirenz, lamivudine, and tenofovir disoproxil fumarate after being diagnosed with HIV. Two years later, he experienced progressive enlargement of the bilateral breasts but did not report the symptoms to his physician. After 6 years of ART, the patient developed grade III gynecomastia in both breasts, accompanied by significant psychological distress, prompting a visit to the breast surgery department. After excluding other potential drug adverse effects or underlying diseases, the patient’s gynecomastia was attributed to efavirenz. Owing to severe enlargement of the breasts, the patient underwent bilateral mastectomy, successfully recovered and was discharged from the hospital. Postoperative follow-up revealed significant improvement in the patient’s anxiety and depressive symptoms.

**Conclusion:**

Gynecomastia represents a nonnegligible adverse effect of efavirenz that clinicians must recognize and manage promptly. Prior to initiating efavirenz therapy, it is imperative to thoroughly inform patients about potential adverse reactions, emphasizing the importance of seeking medical attention promptly should any side effects arise. Early detection and management of gynecomastia can mitigate its psychological impact and ensure continued adherence to ART.

## 1 Introduction

The latest statistical data released by the World Health Organization in July 2024 highlight the alarming global situation of HIV/AIDS, with the number of affected individuals reaching approximately 39.9 million (1). Antiretroviral therapy (ART) works by inhibiting key enzymes in the HIV replication cycle and blocking the entry of HIV into cells. Efavirenz, a non-nucleoside reverse transcriptase inhibitor, serves as a crucial component of first-line ART owing to its exceptional antiviral efficacy (2). According to the literature, the incidence of gynecomastia in HIV-positive male patients receiving efavirenz-based ART is 5%. Among them, over half of the male patients with gynecomastia experience psychological distress, and one-third of these patients do not disclose their gynecomastia to their physicians (3). This paper presents a case of an HIV-positive male patient who developed bilateral grade III gynecomastia while undergoing efavirenz-based ART. Breast ultrasound revealed breast hyperplasia. No other etiological factors were identified in either the imaging or laboratory findings. The patient ultimately underwent bilateral mastectomy, with good postoperative recovery and marked improvement in psychological distress, such as anxiety and depression. Through an analysis of this case, we aimed to provide clinicians with more comprehensive information on efavirenz treatment to better manage patient adverse reactions, enhance treatment adherence, and improve quality of life.

## 2 Case presentation

**Figure 1:**
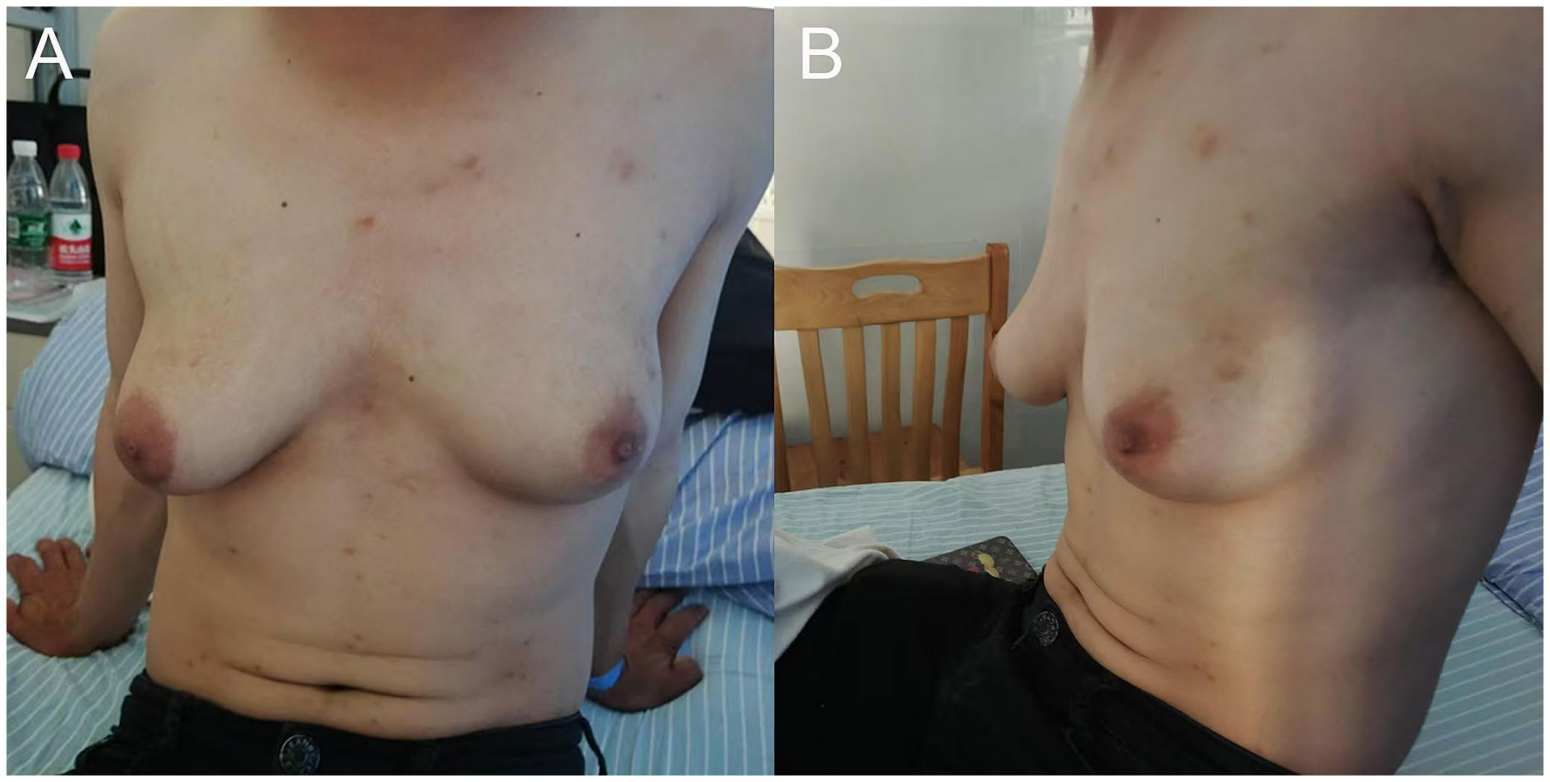
**(A)** Front view and **(B)** side view of the patient demonstrating grade III gynecomastia, with more pronounced development on the right side compared to the left.

A 29-year-old male patient was diagnosed with HIV and subsequently initiated ART consisting of efavirenz (600 mg orally once daily), lamivudine (300 mg orally once daily), and tenofovir disoproxil fumarate (300 mg orally once daily). Two years into treatment, the patient developed gradual bilateral gynecomastia accompanied by mild breast pain, without nipple discharge or other discomfort. During this period, the patient did not seek medical advice for this condition, and the ART regimen remained unchanged. Six years after initiating ART, the patient’s breasts had enlarged to the extent that they were difficult to conceal with clothing, resembling female breasts, which led to feelings of inferiority, anxiety, and other emotional disturbances. The patient presented to our hospital’s breast surgery department, where a physical examination revealed severe bilateral gynecomastia, with the right side being larger than the left (Figure 1).

**Figure 2:**
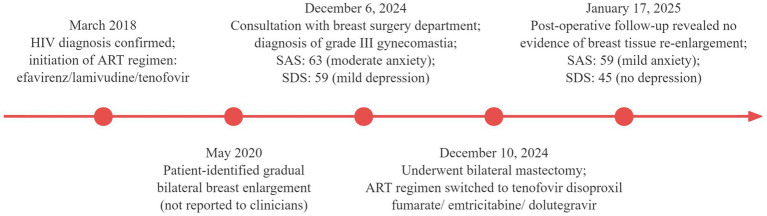
Timeline of key diagnostic and therapeutic events in the patient’s clinical course.

No palpable masses were found, and there was no nipple discharge or bleeding. Scores on the Self-Rating Anxiety Scale and Self-Rating Depression Scale were 63 (indicating moderate anxiety) and 59 (indicating mild depression), respectively. The HIV antibody test was positive, whereas the HIV nucleic acid test was negative. Liver function, renal function, routine blood tests, sex hormone levels, tumor marker levels, and other laboratory test results were normal. Breast ultrasound revealed glandular echoes around the nipples on both sides of the chest wall, with no mass echoes, suggesting male breast development. Pituitary magnetic resonance imaging was normal. The patient underwent bilateral mastectomy under general anesthesia. The resected specimens included left breast tissue measuring approximately 10 × 8 × 4 cm and right breast tissue measuring approximately 11 × 10 × 5 cm. Histopathological examination confirmed bilateral gynecomastia. Following consultation with an infectious disease specialist, the ART regimen was switched to emtricitabine (200 mg orally once daily) combined with dolutegravir (50 mg orally once daily) and tenofovir disoproxil fumarate (300 mg orally once daily). The patient recovered postoperatively and was discharged. At the one-month postoperative follow-up in the breast surgery outpatient clinic, physical examination revealed no bilateral breast enlargement. Scores on the Self-Rating Anxiety Scale and Self-Rating Depression Scale were 59 (indicating mild anxiety) and 45 (indicating no depression), respectively, both showing improvement from preoperative levels. Key events throughout the patient’s diagnostic and treatment course are presented in Figure 2.

## 3 Discussion

Gynecomastia is a benign condition characterized by the proliferation of male breast tissue, clinically manifested by bilateral or unilateral progressive enlargement of the breasts, palpable masses beneath the areolas, occasionally accompanied by breast pain or tenderness, and rarely by galactorrhea (4). Its etiology can be categorized into physiological, pathological, pharmacological, and idiopathic types (5). A retrospective study by Mikkel et al. on 786 patients with gynecomastia revealed that approximately 17% of cases were drug-induced (6). According to the literature, approximately 5% of patients taking efavirenz develop gynecomastia (3).

Upon admission, the patient underwent hormonal tests, breast ultrasonography, and pituitary MRI, which ruled out pathological causes. On the basis of the Naranjo Adverse Drug Reaction Probability Scale (7), the following scores were assigned: previous literature reports of gynecomastia after efavirenz use (+1); onset of gynecomastia following efavirenz administration (+2); absence of other causes for gynecomastia (+2); and presence of objective evidence confirming this adverse reaction (+1). The total score was 6, indicating a probable relationship between efavirenz and the reported case of gynecomastia. Possible mechanisms include the following: (1) Reduced androgen synthesis. CYP17A1 and CYP21A2 catalyze androgen synthesis. Efavirenz inhibits CYP21A2 and CYP17A1, leading to decreased androgen synthesis and an imbalance in the androgen/estrogen ratio, thereby inducing breast development (8–10). (2) Increased estrogen production. Immune reconstitution inflammatory syndrome is a clinical syndrome characterized by fever, latent infections becoming active, and exacerbation or worsening of existing infections during the recovery of immune function after ART in AIDS patients. In IRIS, an increase in CD4+ T lymphocyte counts results in elevated production of inflammatory cytokines such as interleukin-6 (IL-6). Increased IL-6 enhances aromatase activity in breast tissue, which converts androgens into estrogens, stimulating breast tissue proliferation (11, 12). (3) Direct activation of estrogen receptors. Efavirenz directly binds to estrogen receptor-α, promoting breast tissue proliferation (13).

According to the literature, 75% of cases of gynecomastia occur within 2 years of initiating efavirenz therapy, with 58% of patients experiencing bilateral breast development. When this adverse effect emerges, prompt discontinuation of efavirenz and guideline-directed transition to alternative ART regimens is imperative (14). After efavirenz was discontinued, 83.5% of the gynecomastia cases regressed, with an average regression time of 3 months. Most patients with gynecomastia feel embarrassed and inferior, and approximately one-third do not report breast development to healthcare providers, missing the opportunity for early diagnosis and treatment (3). The male patient reported in this case did not consult a physician about his bilateral breast development for 4 years. He only sought care from a breast surgery department after his bilateral breast development reached Simon classification grade III gynecomastia, accompanied by moderate anxiety and mild depressive symptoms. In early-stage gynecomastia, before irreversible fibrosis and hyalinization of glandular tissue occur, discontinuing efavirenz may lead to improvement. However, after 4 years of disease progression culminating in Simon classification grade III gynecomastia, the patient’s glandular tissue had likely developed irreversible fibrosis, making surgical resection the definitive intervention for addressing both physical and psychological morbidity (5). Due to the relatively underdeveloped economic and medical conditions in our region, such severe cases of gynecomastia are rare. During treatment, since the patient had no objection to nipple-areola resection, preservation was not attempted. Although the patient reported fair satisfaction with the surgical outcome, performing bilateral mastectomy without preserving the nipple-areola complex was suboptimal. Published literature indicates that surgical techniques such as the circumareolar incision-based bilateral pedicle vertical flap mastectomy and vacuum-assisted mastectomy combined with power-assisted liposuction can achieve favorable therapeutic and esthetic outcomes for such patients (15, 16).

This case report has several limitations. First, as a single-case study, it lacks statistical power and generalizability. The retrospective nature of the data, derived from medical records, is less systematic than a prospective design. Notably, hormonal/metabolic data at gynecomastia onset were unavailable; normal preoperative levels do not rule out transient imbalances. Additionally, short follow-up after efavirenz discontinuation and surgery prevents assessment of long-term efficacy and recurrence risk. Despite thorough workup, multifactorial contributors to gynecomastia cannot be conclusively excluded.

Breast development in HIV-positive male patients during antiviral treatment can exacerbate their psychological burden, affecting their social interactions, daily lives, and adherence to antiviral treatment. Gynecomastia is one of the adverse effects of efavirenz, which clinicians should take seriously and manage promptly. Before initiating efavirenz treatment, patients should be fully informed about potential adverse reactions and encouraged to seek help promptly if they occur, enhancing patient adherence to treatment.

## Data Availability

The original contributions presented in the study are included in the article/supplementary material, further inquiries can be directed to the corresponding authors.
